# Geochemical study of carbonate concretions from the aqueduct of Nîmes (southern France): a climatic record for the first centuries AD?

**DOI:** 10.1038/s41598-019-41620-4

**Published:** 2019-03-26

**Authors:** Yacine Benjelloun, Julie Carlut, Jean-François Hélie, Gilles Chazot, Laurence Le Callonnec

**Affiliations:** 1 Institut de Physique du Globe de Paris, Sorbonne Paris Cité, Université Paris Diderot, UMR CNRS 7154, 75005 Paris, France; 2 Université du Québec à Montréal, Département des Sciences de la Terre et de l’atmosphère et centre GEOTOP, Montréal, H3C 3P8 Canada; 3 Université de Brest, UMR 6538 Géosciences Océan, Institut Universitaire Européen de la Mer (IUEM) Place Nicolas Copernic, 29280 Plouzané, France; 4 Sorbonne Université, CNRS-INSU, Institut des Sciences de la Terre de Paris, ISTeP, F-75005 Paris, France

## Abstract

The first centuries AD in the Mediterranean region have generally been associated with a warm, stable climate. High-resolution sedimentary archives sensitive to local environmental change are needed to switch from this general frame to the regional scale. Similarly to cave speleothems, laminated carbonate deposits can grow in the channels of aqueducts which transported water from karstic springs during the Roman period. The deposits of the aqueduct of Nîmes (SE France) are exceptional since they may represent several centuries of paleoenvironmental record with a seasonal resolution. δ^18^O, δ^13^C and trace elements were measured in three samples from this aqueduct. The comparison of the geochemical signals with the fine texture of the deposits evidenced the seasonal nature of the lamination observed. This allowed us to document the evolution of environment as recorded through the deposit for the period 50–275 AD. The concretions of the aqueduct of Nîmes document rather stable climatic conditions for the first three centuries AD, as well as a local vegetation change possibly linked to an increased in land use.

## Introduction

The recent developments of geochemistry, dendrochronology, and palynology have allowed a better understanding of the European climate for Roman times, which is generally presented as a warm period marked by a strong irregularity after the third century AD^1,2^. In continental areas, the evolution of climate can be retrieved from freshwater carbonate deposits such as laminated tufa growing in caves or rivers^3^. For the historical period, analogous deposits can develop inside anthropic structures used for water delivery, such as the aqueducts built around the Mediterranean during the Roman period^4^. The interest of those deposits was acknowledged early, and several authors used them to estimate the evolution of paleo-flow rates^5^ or distinguish successive phases of the building^6,7^. More recently, these deposits were studied for paleoenvironmental purposes as they couple a good sensitivity to external forcings and a reliable chronology owing to the archaeological context^8^. This context allows, in turn, the discussion of the signals preserved in these deposits in terms of anthropological impacts and historical interactions between environment and societies.

Our study is focused on calcium carbonate deposits sampled at three different locations along the channel of the aqueduct of Nîmes in southeastern France. With macro and microscopical observations, oxygen and carbon stable isotopes analysis, and trace element variations, we discuss the proxies and the climatic component of the textural and chemical patterns observed on these deposits at the inter-annual and sub-annual scales. We also compare the signals recorded by these concretions and interpret them in terms of regional variations and anthropic phenomena.

## The Aqueduct of Nîmes and its Concretions

**Figure 1:**
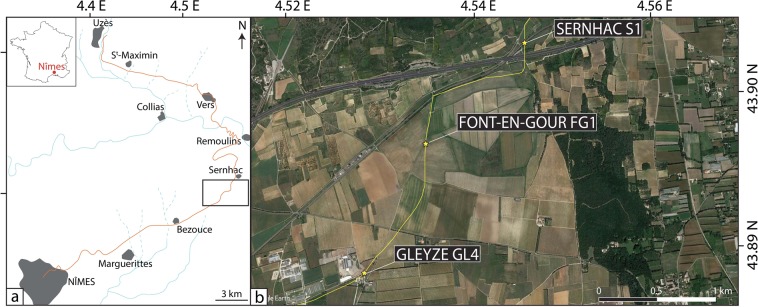
Geographical context of the study. (**a**) Location map of the aqueduct of Nîmes (orange) with the main towns in grey and hydrographic features in blue. (**b**) Location of the three concretion samples. These three locations correspond to underground portions of the aqueduct which could be reached from the surface by road cuts and vertical pits built for maintenance purposes. The satellite imagery was taken from Google Earth (data provider: Image Landsat/Copernicus).

The Roman city of Nîmes was supplied with water thanks to an aqueduct system, made famous by the Pont du Gard monument (Fig. 1a). By contrast with the Pont du Gard, most of the length of the aqueduct system consisted in channels buried under ground. The aqueduct’s sources were located at the fontaine d’Eure (72 m asl) near the town of Uzès, in a limestone massif of Barremian age. From these springs, the water was carried by gravity for 50 km until the city of Nîmes (59 m asl), where it was distributed through different pipes via a *castellum divisorium*. It was built around 50 AD during the reign of Claudius or Nero^6^. After a few centuries of optimal functioning, is started to degrade in the first half of the 3^rd^ century and was then partially abandoned. After trials of rehabilitation between the 4^th^ century and the early 5^th^ century, it stopped working definitively between the 5^th^ and the 7^th^ centuries^6^. Given the karstic nature of the terranes in which the sources are located, the water used by the aqueduct was enriched in dissolved calcium and bicarbonate. As the partial pressure in CO_2_ decreased, the water flow in the aqueduct’s channel led to the precipitation of calcium carbonate concretions on its sides, with a possible role of bacterial activity^9^.

**Figure 2:**
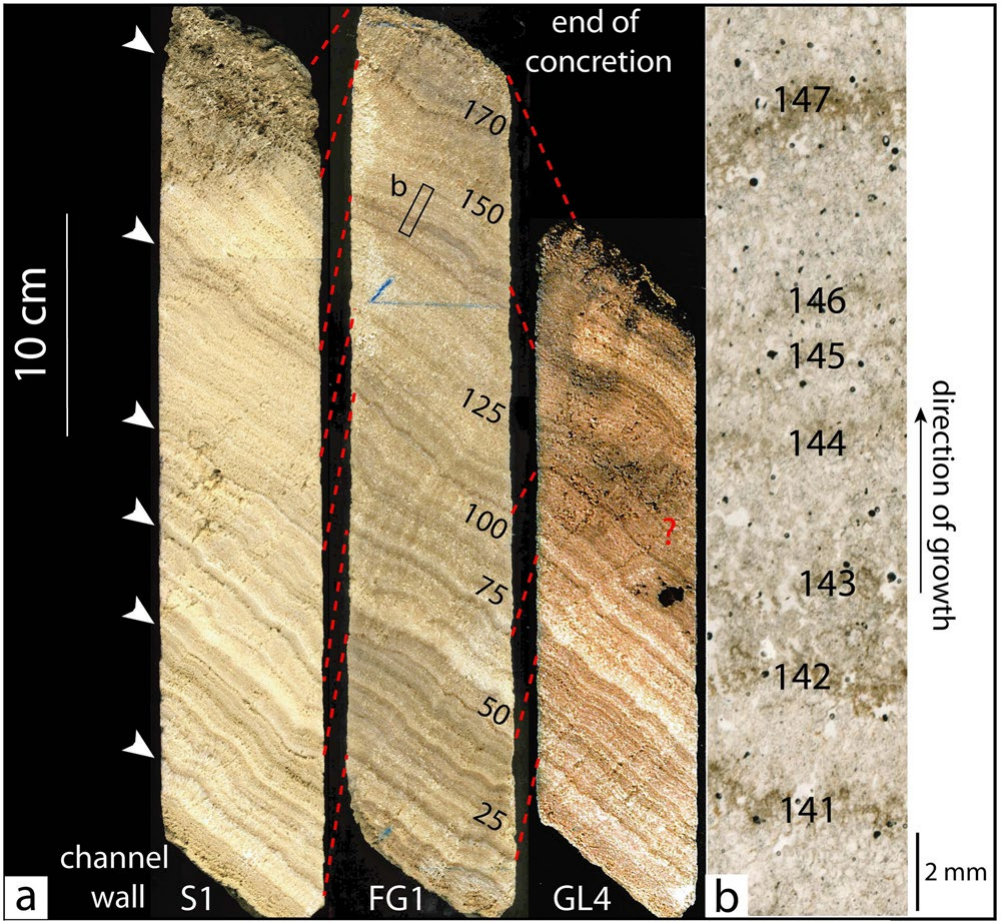
Texture of the concretions. (**a**) The three concretion samples of Sernhac, Font-en-Gour, and Gleyze, from left to right. The number of laminae couplets is indicated on the central sample. The dark porous joints mentioned in the text are located on Sernhac with white arrows. A few remarkable levels are correlated in red between the three samples. Note that the correlation with Gleyze stops after about 15 cm of concretion. (**b**) Example of light-dark alternating laminae. The photograph is a scan of FG1 thin section under transmitted non-polarized light. The numbers written on the dark laminae correspond to the counting.

The concretions analyzed in this study, S1, FG1 and GL4, were sampled using a 7.5 cm diameter drill in October 2000 by the company GEO-TER at three different locations along the aqueduct’s route: Sernhac, Font-en-Gour, and Gleyze (Supplementary Table S1 and Fig. 1b). The deposits are composed of a series of white to brown horizontal bands, parallel to the channel’s wall (Fig. 2a). At the macroscopic scale, the concretions can be divided into two different parts. From the wall, after 2 cm of yellowish porous bands, the first half is characterized by a succession of relatively thick bands of very different colors, ranging from white to dark brown. It is also possible to identify four thin and dark porous joints separating larger clusters of bands. The second half of the concretions is more homogeneously colored and is composed of light brown, thinner strata with little contrast between the bands. Two darker joints are visible in the middle of this part and about 3 cm from the end of the deposit, characterized by high porosity. Most of these textural features can be found similarly on the three concretions sampled, but GL4 is significantly shorter than S1 and FG1. The textures of the three deposits can be correlated well for the first 15 cm, after which the sedimentation of GL4 differs (Fig. 2a).

## Results

### Microscopic observations

**Figure 3:**
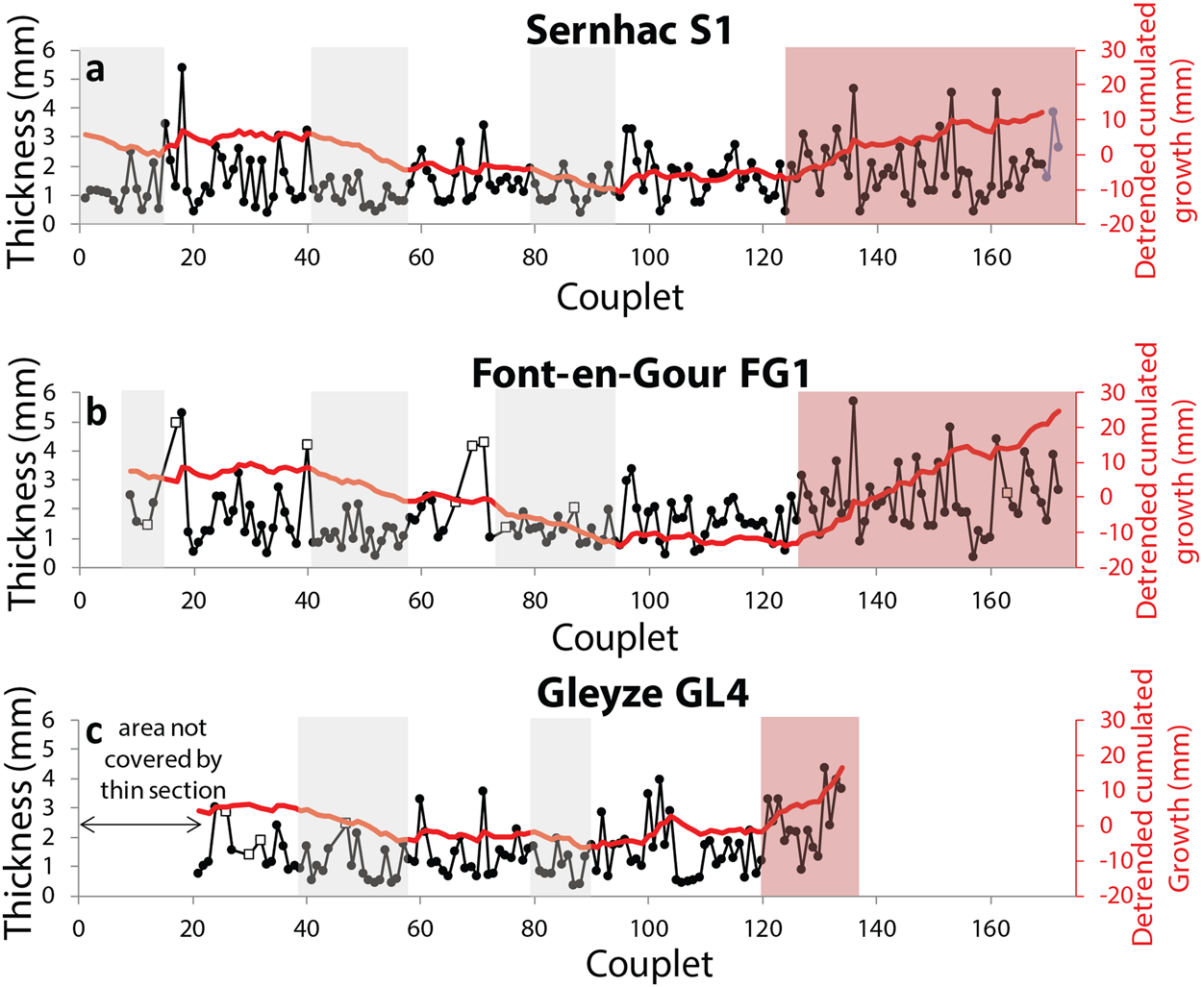
Measured thicknesses of the laminae couplets. (**a**) Sernhac. (**b**) Font-en-Gour. (**c**) Gleyze. The location of the laminae couplet is indicated on the horizontal axis, and its thickness on the vertical axis. The first centimetres of sample GL4 are not included in the thin section used. The succession of couplets can generally be correlated well across the three samples. Sernhac seems the longest and most complete record, and is thus considered as the master curve for the age model used afterwards. The three last values of FG1 were reported on S1 in blue. The red curve corresponds to the subtraction between the thickness curve and the long-term growth trend. The remarkable phases of slower and faster growth visible across the samples are indicated in grey and red respectively (see also Supplementary Fig. S1). The white squares correspond to successive couplets that could not be distinguished on some samples. The entire dataset can be found as Supplementary Table S2.

Successive laminae, alternatively bright and dark, of millimetric thickness are observed throughout the concretions (Fig. 2b). The bright, transparent laminae observed in thin section are brown and translucent to the naked eye. The dark, opaque laminae in thin section present various shades on the sampled concretion, from white to brown, but are also opaque. A similar structure is well known for natural river travertines and speleothems. Contrary to river travertines, the aqueduct concretions in this study do not display vegetal remains or cyanobacterial structures and the lamination generally looks regular under the microscope. In many cases, it has been shown that this lamination in natural and archeological tufa is annual, and the dark-bright alternation linked to the seasonality of precipitations^4,10^. This color alternation has generally been associated with variations in crystalline properties. The dark and bright laminae correspond to dominantly micritic and sparitic layers, respectively, although this pattern may vary significantly between different layers and at different locations of the same layer^4,5^. We used color as the main criterion to distinguish successive laminae couplets. The number of dark-bright laminae couplets was counted, and the thickness of each laminae couplet was measured (Fig. 3). On average, the laminae couplets of the three concretions are of similar thickness, around 1–2 mm. Several observation biases need to be considered. (1) In some areas, the contrast on the thin section is low and makes it hard to identify the dark laminae. This can lead to identify a succession of several couplets as one couplet. Some remarkable thickness values, over 5 mm, may be explained by this. (2) It has been reported that dark bands linked to fulvic and humic enrichments may appear inside a bright lamina^4,11^. These dark bands unrelated to seasonal variations are often difficult to differentiate from “true” dark layers separating successive couplets. This can lead to an overestimation of the couplets counted. These two biases have antagonistic effects and are likely to partly compensate each other. Based on our observations, an uncertainty of ±15 laminae couplets, corresponding to ~10% of the total number of couplets, is a conservative estimate.

For the majority of couplets, the bright lamina is much thicker than the dark one. This suggests that the bright laminae develop during periods characterized by a sufficient water flow and well mineralized water. On the contrary, the finer dark laminae are supposed to correspond to periods of weaker mineralization, during dry and hot months when the water is enriched in detrital and organic elements^4^.

Using linear regression, we obtained average growth rates of 1.75, 1.78 and 1.58 mm/couplet for S1, FG1 and GL4 respectively. Despite the relatively high variability in individual laminae thickness, the very high correlation coefficients (0.9956, 0.9865 and 0.9924 respectively) of the linear regression between couplet number and thickness indicate that growth is rather stable along the concretions. The smaller total thickness of GL4 is also associated with a smaller number of couplets. We also note a discrepancy between GL4 and the two other concretions after couplet 105, which is followed by a series of very thin laminae in GL4 (Fig. 3). Despite these differences, the interannual evolution of growth rate is very similar across the three concretions (Fig. 3 and Supplementary Fig. S1). We can especially note two phases of slower growth for couplets 40–60 and 80–100, and a growth increase at the end of the deposit after couplet 125. This episode is also visible for GL4 after couplet 120 (Supplementary Fig. S1).

### Oxygen and carbon stable isotopes

**Figure 4:**
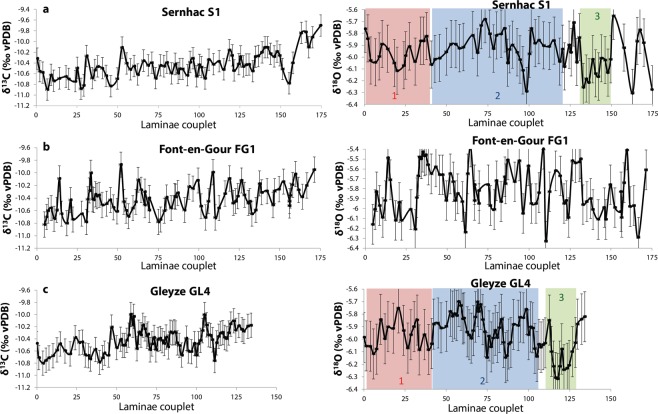
Temporal evolution of δ^13^C (left) and δ^18^O (right), for the three concretions Sernhac, Font-en-Gour and Gleyze (a, b, c respectively). The color bars refer to the three common intervals between S1 and GL4 discussed in the text. The uncertainty on the isotopic ratios is 0.2‰. Two outliers were removed each for GL4 and S1. The entire dataset can be found as Supplementary Table S3.

The overall variations of δ^18^O and δ^13^C are very limited, around 1‰, and in an identical range of values for the three concretions (Fig. 4 and Supplementary Fig. S2). The δ^13^C varies between −10 and −11‰ and the δ^18^O between −5.5 and −6.6‰. These small amplitudes of variation can be explained by the underground location of the channel which strongly attenuates temperature variations and isotopic composition of water. We do not observe systematic correlation between δ^18^O and δ^13^C signals for GL4 and S1 (R^2^ of 10^−2^ and 2.10^−4^ respectively), which suggests that fractionation occurred at equilibrium. For FG1, a stronger correlation (R² = 0.3) is obtained (Supplementary Fig. S3).

A common feature for the three concretions is a long-term similar trend of δ^13^C with a slow increase from −11 to −10‰ (Fig. 4). This trend is not regular but rather consists in several “steps”. This is especially visible for S1 and GL4. The period 0–45 is stable, followed by a rapid increase and another stable period until couplet 105, the ends of both records are marked by another increase of δ^13^C.

The δ^18^O series do not present long-term trend and display small amplitude variations (Fig. 4). The signal obtained for the three concretions differs and in details, they cannot be matched. We still tentatively identified three intervals showing similarities between S1 and GL4:

- The initial interval before couplet 30 corresponds to intermediate values around −6‰.
- The next interval starts between couplet 30 and 40 and shows a trend to higher values around −5.8‰. Some significant negative peaks are visible (e. g. at 100 for S1, 75 and 105 for GL4). A general decreasing trend is observed between 70 and 120, although the interval is shorter for GL4.
- The interval 130–150 is marked on S1 by lower values around −6.2‰. For GL4 this is seen between 113 and 128.

**Figure 5:**
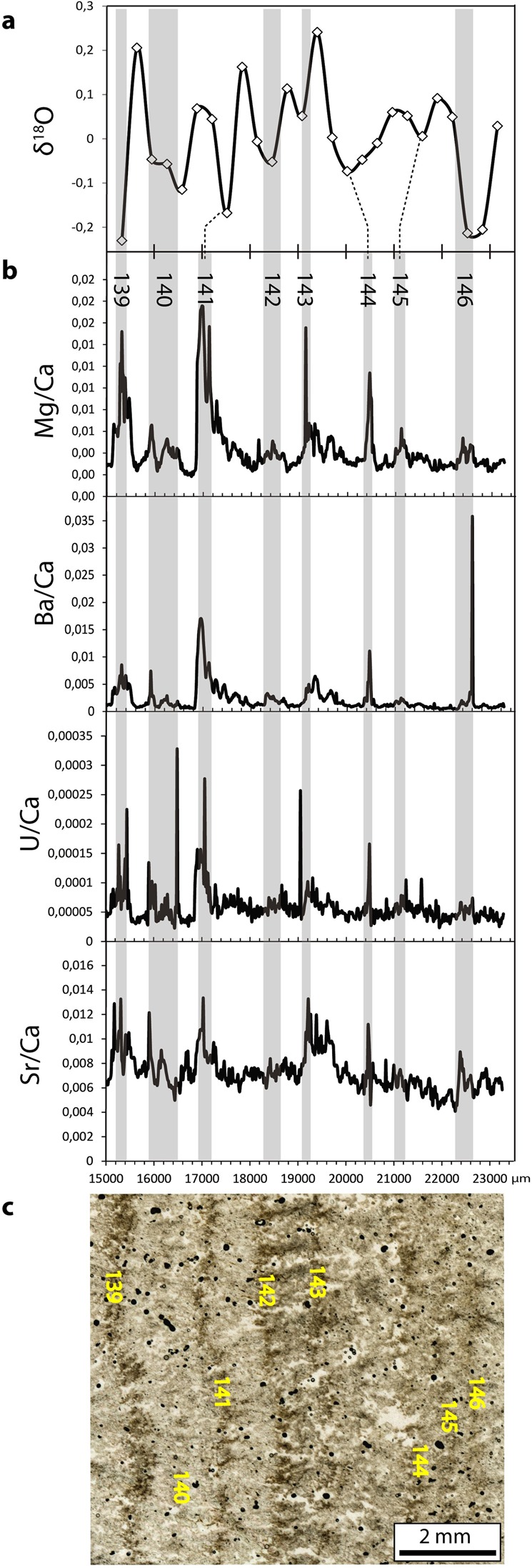
Relation between the geochemical signals and the fine lamination of the concretion S1. (**a**) δ^18^O signal. The δ^18^O data were obtained through micromilling. The interannual trend was modelled by a 2^nd^ order polynomial function and removed. (**b**) Trace element signals. (**c**) Microstructure. The thin section was scanned under transmitted non-polarised light with a resolution of 2400 dpi. We observe a good correlation between the δ^18^O minima, the trace elements peaks and the dark laminae.

At a finer scale, we can observe a first order correlation between the dark/bright laminae alternations and the δ^18^O oscillations. The dark laminae seem associated with minima of δ^18^O (Fig. 5a).

### Trace elements

The trace element composition of the samples varies following the couplets. The darker laminae are generally enriched in trace elements, while the bright laminae show lower values (Fig. 5). Such observations have already been made on other archeological sinters^4,8,11^. This correlation is especially clear for Mg and Ba and less visible for U and Sr depending on the laminae (Fig. 5). At a finer scale, the correlation between elements is variable but most of the major peaks visible correlate well, albeit a few exceptions such as the very high Ba values of couplet 146 (Fig. 5) which are not mirrored by the other elements.

**Figure 6:**
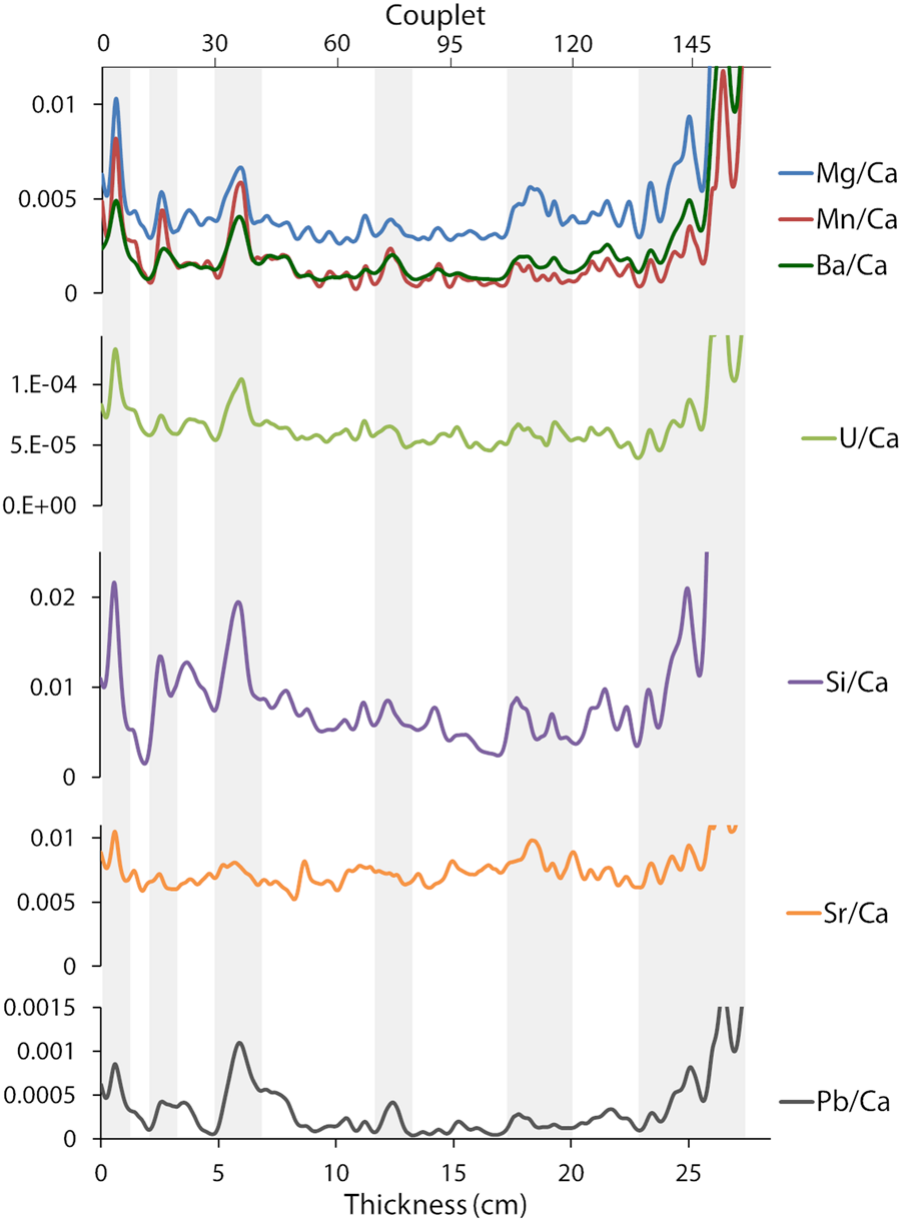
Smoothed variations of trace elements measured on Sernhac deposit. The smoothing was done with PAST software^31^ using a spline function with a smoothing value of 8.5. The raw data are visible on Supplementary Fig. S4. The very end of the concretion gives unrealistic values due to a lower calcite content.

At a longer time scale, four intervals with contrasted variations can be observed (Fig. 6). Between the channel wall and couplets 50 and after couplets 135, the highest values are recorded in all measured elements with the exception of Sr. By contrast, the interval between couplets 50 and 105 is associated with the lowest values and lowest variations for all elements (once again Sr behaves differently). Between 105 and 135, slightly higher values are found for Mg and Ba while variations in other elements are not significantly different from the previous interval.

## Discussion

### Annual signal in the laminae couplets and duration

The region of Nîmes in southern France has been characterized since 5000 years BP by a Mediterranean climate, with a marked wet season during winter and spring and summer droughts^12^. It has been shown that the development of natural travertines in southeast France follows the seasons, with low growth in winter due to poorly mineralized waters, high growth in spring and reduced growth in summer and fall^13^. In archaeological carbonates in Ostia, a short-term signal in trace elements linked to seasonal oscillations was identified and correlated to the dark-bright laminae alternations^8^. In the present study, the comparison of the trace element signals with the lamination (Fig. 5b,c) leads to a similar result: the peaks in most elements correlate well with the dark laminae. This is consistent with an increased concentration of elements during the dry and hot season due to higher dust content and/or longer water residence times in the karst. Biological activity, such as the growth of biofilm or fungus during the hot season, may also be responsible for higher concentration in detrital elements in the dark laminae^4^.

Micromilling results show that the dark laminae and trace elements peaks correlate with relative minima of the δ^18^O interpreted as temperature maxima (Fig. 5). Similar observations were made in previous studies^4^ and strengthen the interpretation of the couplets as yearly deposits. However, occurrences of irregular layering were evidenced in a concretion from the aqueduct of Béziers^11^. In particular, packages of brown layers were explained by multiple high discharge events recorded during the same rainy season. Such packages were sometimes observed in our samples and are treated as uncertainty added to the laminae numbers.

Using the laminae couplets as yearly indicators, it is possible to put the textural and geochemical observations in a relative temporal frame. In that hypothesis, the concretions of Sernhac and Font-en-Gour give access to around 175 years of functioning of the aqueduct. It is probable that their growth was not continuous because of flow interruptions linked to maintenance works. Therefore, the functioning duration given by the sampled concretions is likely to underestimate the real duration. The almost two centuries of functioning could correspond to the “optimal” period of operation for the aqueduct. When compared to Sernhac and Font-en-Gour, Gleyze recorded 40 years less. If a date of 50 AD is taken as a start, the samples recorded the functioning of the aqueduct until at least 225 AD.

### Identification of growth interruptions in Gleyze sample

GL4 is shorter than the other samples and we estimated that around 40 years of record are missing in the concretion. It is thus more difficult to correlate the signals of GL4 with those of the two other samples. However, several similarities were observed in the growth rate and isotopic compositions, especially with S1. Based on these similarities, the parts of S1 which are missing in GL4 can be pointed out. The comparison of the laminae thickness curves shows that the interval 0–90 is apparently well preserved in GL4 (Fig. 3). A gap of at least 5 years can be inferred from the slight shift of the interval between the second low growth period and the final high growth period. We can therefore assume that the decorrelation of GL4 started during this interval. Finally, a significant part of the interruption occurred during the “high growth” period, which lasted 50 years in S1 and only 10 years in GL4.

Using the δ^18^O signals, the decorrelation of GL4 may be visible in the year 93: the δ^18^O drops significantly in S1 whereas it keeps the same range in GL4 (Fig. 4). For the following years, it is possible to correlate both records if the GL4 curve is shifted 20 years forward. This way, we identify the low ratio intervals mentioned above (interval 3). In this scenario, we propose at least a first interruption between 90 and 110 and another interruption in the last 20 years. The δ^13^C curves are compatible with this scenario: the first “jump” is visible in S1 and GL4 around year 50, and the second one happening around year 120 in S1 may be matched with a peak in GL4 in year 105. Obviously, the reality may be more complex, with a higher number of shorter interruptions that we cannot resolve with our datasets but we clearly identified two periods of time which are not recorded in Gleyze. The morphology of Gleyze deposits on the channel walls exhibits a regressive evolution with a reduced thickness at the top^14^. This indicates a lowering of the water level downstream of Font-en-Gour in the final period of sedimentation.

The existence of these anomalies in the concretions of Gleyze suggests perturbations starting after the middle of the second century AD. This could result from leakage following a progressive decay, or a tectonic deformation^14,15^. Anthropic interventions for maintenance, cleaning, or water withdrawals, are other possibilities. Given that traces of degradation are not clear in the section studied, we favour an anthropic origin. The aqueduct’s section between Font-en-Gour and Gleyze crosses the large agricultural plains that stretches southeast of the Nîmes garrigue and west of the Rhone river. This area has likely been exploited since the Roman period, and water withdrawals from the aqueduct, for example to supply hydraulic mills^6,9^, can be proposed to explain the anomalies in the concretions. The underlying causes of these withdrawals are more difficult to establish but may include climatic degradations (colder and/or dryer episodes), or agricultural expansions linked to economic and demographic growth.

### Remarkable variations along the growth axis

We identify three episodes of lower growth rate in the concretions:

- (1) The first 7–8 years of the record in Sernhac correspond to the first immersion of the conduits. Regulation and adjustments were likely responsible for a reduced flow rate. Reaction with the waterproof has also been proposed for the lower growth often observed at the base of aqueducts concretions^16^.
- (2) During years 90–108 AD (taking 50 AD as a start date) and (3) during 125–145 AD, these low growth intervals are roughly observed in the three records, although some years are not recorded in Gleyze.

By contrast, the last 50 years corresponds to a higher growth rate for S1 and FG.

**Figure 7:**
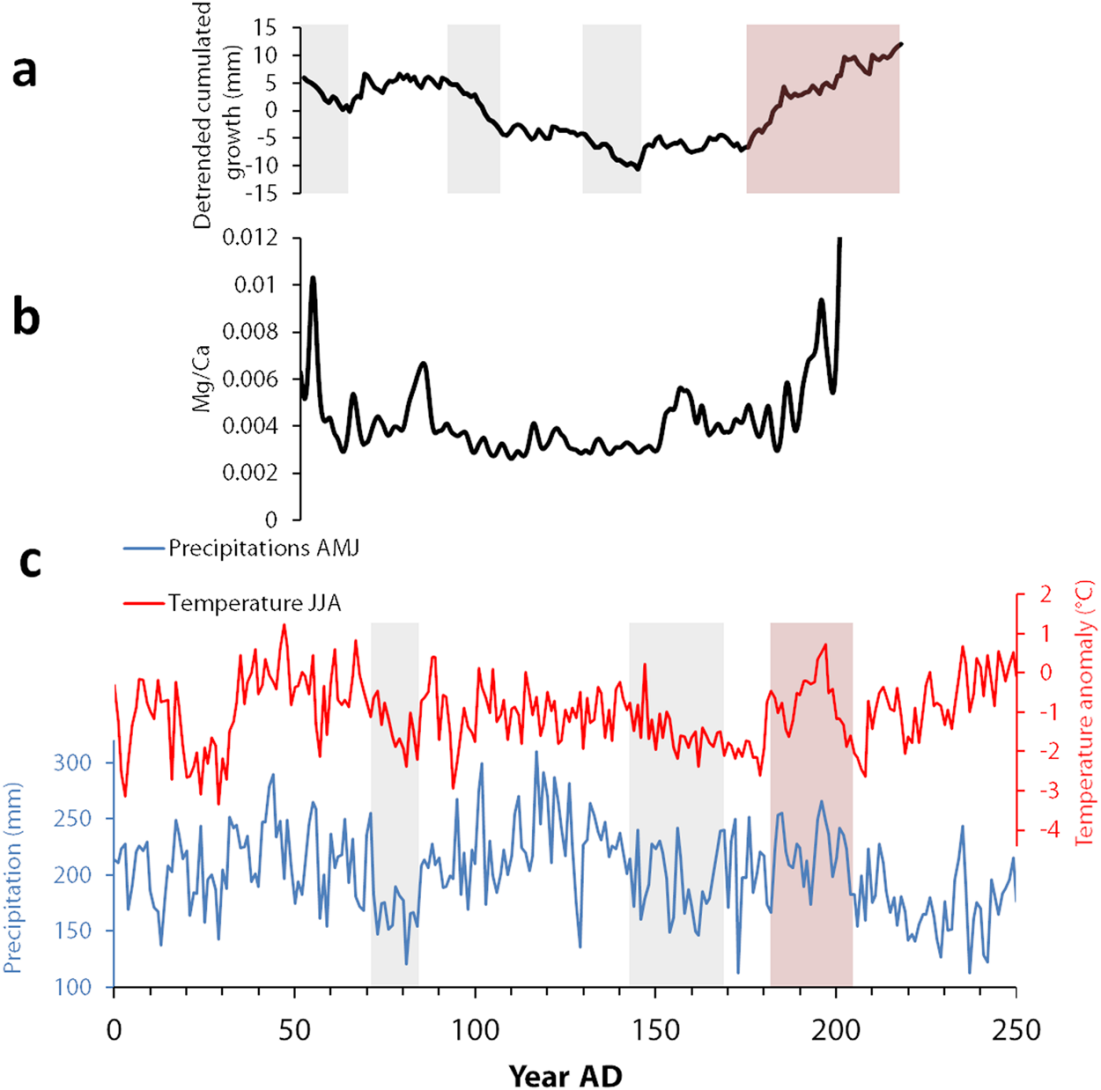
Relation between the historical climatic variations and the concretion record. (**a**) Detrended growth curve of S1. The remarkable slow and fast growth periods are shown with grey and red boxes respectively. (**b**) Smoothed Mg/Ca curve obtained on S1. The variations observed for the other elements measured are generally correlated with Mg/Ca, with the notable exception of Sr/Ca (Fig. 6). (**c**) Reconstructed temperature and precipitation variations for the period of the concretions’ growth^2^. The grey boxes and the red box locate respectively the periods of low precipitations and high temperature-precipitation mentioned in the discussion.

Calcite growth in travertines depend on several parameters, such as the chemical composition of water, the amount of rain, the partial pressure in CO_2_ in the karst, and local hydrodynamic factors^17^. Several studies suggest that a decrease in the growth rate indicates lower flow rates and/or colder conditions^17,18^. For the aqueduct concretions, we expect growth to be high during spring and early summer, when water is abundant and temperatures rise^13^. A deficit in precipitation and/or lower temperatures during the spring could then be associated with thinner laminae. A 2500 years region-wide, year to year AMJ (April-May-June) precipitation curve was compiled for Northern France^1^ (Fig. 7c). A strong correlation between Southern and Northern France AMJ precipitations from the 1^st^ century BC to the 2^nd^ century AD was demonstrated using oak tree rings variation curves^19^. This allows us to use the reconstructed (AMJ) precipitations over time for Northern France as a proxy for spring precipitations around Nîmes for the 0–200 AD interval. Two pronounced minima in the precipitation record corresponding to several AMJ years with dryer conditions are clearly isolated around roughly 70–85 AD and 140–170 AD. Both correspond also to colder summers^1^. Thus, it appears that decades of deficit in precipitation and lower summer temperature could have affected the region of Nîmes at the end of the first century AD and middle of the 2^nd^ century. Two minima were also observed in the growth rate of the concretions, although not strictly correlated in time with the two precipitation minima (90–108 AD instead of 70–85 AD and 125–145 AD instead of 140–170 AD respectively). Given the dating uncertainties on the initiation of the aqueduct’s use, the tree rings dating error bars, the uncertainties in the laminae counting and possible missing years in our records, it is not possible to discuss these timing differences accurately. However, the occurrence of two intervals of low growth rate in our concretions gives credibility to the possible record of decades of dryer conditions reported around the same time. Reciprocally, the warmer and rather wet period deduced from tree rings between 175 and 210 AD is mirrored by higher growth rate in the concretions at the same time. These episodes could have impacted the karst systems and *in fine* the growth of the aqueduct concretions. Further data are of course mandatory to test the influence of local factors in the possible time lag between precipitation changes and karst response, or anthropic factors such as regulation and/or upstream withdrawal, which can strongly affect the deposits.

Carbonate δ^13^C has two main sources: the carbon of the bedrock and the organic carbon present in the soil above the karst system. The final isotopic composition measured in the carbonate deposit thus depend on several factors: vegetation cover, residence time of water in the soil and in the karst, isotopic signature of the bedrock^20^. The water DIC at the source and its evolution in the aqueduct, and the biological activity in the aqueduct (e.g. presence of biofilms) can also influence δ^13^C. Degassing of CO_2_ linked to variations in the water discharge rate can have a strong, positive impact on δ^13^C^4^. The carbon signal of the concretions mainly shows a general positive trend with small amplitude noise. This trend may indicate a change in the vegetation cover surrounding the karst surface. This is consistent with a local increase in land use during the first centuries AD. Agriculture tends to favour C4 species, which have a higher signature in carbon-13^21^. Such regional changes have been documented by previous studies^22^ which observed a phase of environmental degradation in the lower Rhone valley during the 2^nd^ century AD linked to widespread deforestation. The irregular pattern observed, with rapid “jumps” separating longer, stable periods, might indicate that this expansion of land use was not realized progressively but with punctual phases of cultivation. The expansion of cultivated terrains can also be put in relation with the water withdrawals revealed by the deposit in Gleyze especially after couplets 105–110.The city of Nîmes became a regional capital during the 2^nd^ century AD and subsequently expanded over its countryside^23^.

Carbonate δ^18^O depends primarily on two factors: temperature and the isotopic ratio of the water from which the CaCO_3_ precipitated. Kinetic processes occurring during precipitation or evaporation can also play a role^20^. In closed conduits, water evaporation is generally limited and is not likely to significantly influence the δ^18^O signal^4^. Aqueduct channels are shallowly buried structures that can experience seasonal temperature changes, though minor^4^. The study of other sinter deposits from southwest Turkey and southern France evidenced that the bright laminae, which correspond to the winter rains, were associated with high δ^18^O values^4,11,24^. The same authors propose that the seasonal changes in the isotopic composition of rainfall cannot explain this periodicity in the isotopic signal because most rainfall in these regions occurs in winter, and the mixing in the karst is likely to remove this seasonal pattern from the spring water^4,25^. They favour a temperature-dependent fractionation: the higher δ^18^O values result from the lower winter temperatures. This association between high δ^18^O values and the bright laminae was found in our sub annual sampling (Fig. 5). In our case, the variation range corresponds to a maximum temperature variation slightly above 2 °C^26^, if the precipitation is in equilibrium with water.

At a longer time scale, if we use S1 signal as master curve, we observe a rather stable phase during the first 80 years (50–130 AD) with a positive trend (Fig. 4). This is followed by more significant and irregular variations, with a low ratio episode between 180 and 200 AD. When comparing this signal with the reconstructed temperature curve^1^, it seems that the interannual δ^18^O variations are not primarily driven by the summer temperatures. Moreover, the discrepancies with FG1 signal suggest that local factors, such as vegetation growth and biological activity, may play a significant role. This limits the possibility to interpret these signals in terms of regional phenomena.

**Figure 8:**
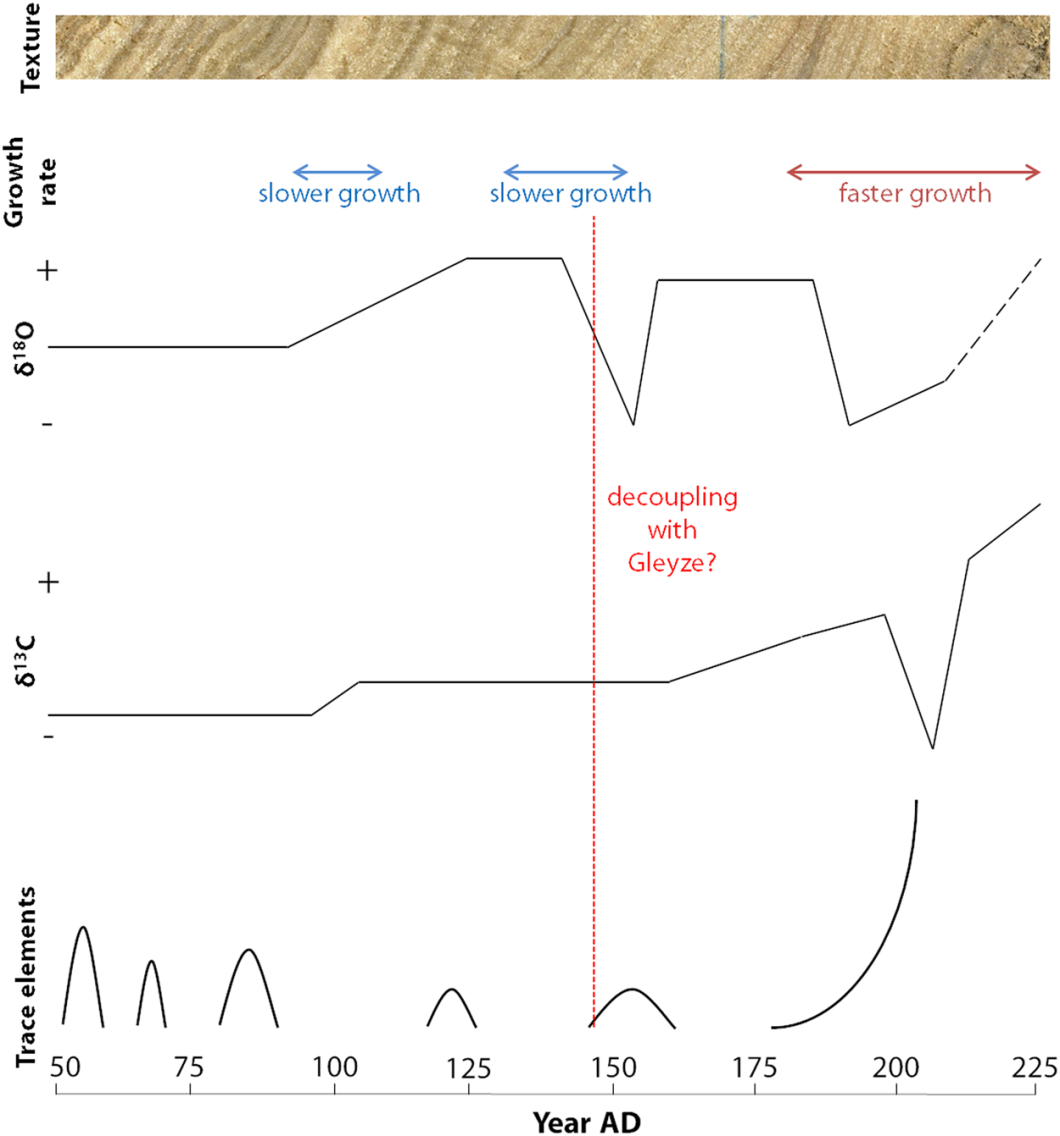
Comparative summary of the variations of the different proxies during the growth of the concretion. We assume that the aqueduct started working around 50 AD. From top to bottom, we present the macroscopic texture of the concretion (the photograph was taken on FG1), the periods with anomalous growth rate, the simplified variations of the isotopic ratios, and the main peaks of trace elements observed. We also indicate in red the time when the isotopic signals of GL4 decorrelate from S1.

In general, we observe that the main trace element peaks happen during periods of faster growth and correspond to dark, porous layers (Figs 6 and 8). The crystal growth rate of calcite can influence the incorporation of trace elements. For instance, high growth rates have been associated in previous studies with higher Sr and Ba values^27^. At the first order, an overall correlation between most trace elements variations and reconstructed summer temperatures is visible (Fig. 7). The relatively higher trace element values observed during the first decades and in the end of S1 correspond to warmer phases. Inversely, the stable, lower values measured in the middle of the concretion correspond to lower summer temperatures. The positive effect of temperature on growth rate, and of growth rate on trace elements values has also been observed in previous studies of cave speleothems^28^. However, trace elements variations can also be linked to local processes in the aqueduct. The very high values at the end of the deposit are associated with brown and porous layers enriched in detrital particles, which probably results from the lack of maintenance and degradation of the building in the 3^rd^ century AD, when the city of Nîmes started to decline^23^.

## Conclusions

Our study is the first application of high-resolution, coupled textural and geochemical analyses on the concretions from the aqueduct of Nîmes. Given the complex context of these deposits, the multiple factors controlling the proxies, and the absence of a modern analogous system, their interpretation remains delicate. However, we show that these deposits present both a long-term environmental signal and a short-term seasonal signal, which allows us to build a chronological frame of yearly resolution. Considering a date of 50 AD for the beginning of our concretions and assuming a relatively continuous record, at least for Sernhac and Font en Gour, the analysed concretions provide a paleoenvironmental archive covering the period 50–225 AD.

The variations of growth rate and trace elements were compared with recent reconstructions of past temperatures and precipitations. The similarities observed suggest that these proxies may be primarily driven by these climatic parameters. In our case, higher trace elements values are consistent with warmer episodes around 50–70 AD and in the end of the second century, the latter also corresponding to higher growth rates. In addition, two low growth episodes may be related to dryer conditions in the end of the first century and in the middle of the second century AD.

A drop of the water level in the channel was deduced from the different morphology of Gleyze sample. Water withdrawals of human origin for agricultural or industrial purposes is a possible scenario. This social pressure on the aqueduct water is corroborated by the δ^13^C signal which suggests a vegetation change in the environment of the karst feeding the aqueduct. This might reflect the increased agricultural activities during the Roman period. At the end of the concretion the isotopic ratios and trace elements show unusual variations which may reflect the degradation of the aqueduct system at the beginning of the 3^rd^ century AD.

Finally, the δ^18^O variations suggest stable conditions until the middle of the 2^nd^ century AD. This is consistent with the historical and environmental datasets compiled over the Mediterranean region^29^. However, while the δ^18^O signal probably includes a climatic component, the differences observed between the three concretions and the absence of a clear relationship with the other data presented make us sceptical about its interpretative value. Other contemporaneous datasets are critical to assess the relative importance of regional versus local parameters in the interpretation of δ^18^O from such concretions.

While our study confirms again the value of archaeological carbonates as paleoenvironmental archives, we also evidenced that three concretions from the same aqueduct can present significant geochemical and morphological differences. This emphasizes the importance of studying multiple samples in one site. In this regard, growth rate and trace elements provide very valuable information. Recovering climatic information from these records remains challenging as human action can obviously bias the record. Additionally, sensitivity to climatic forcing is strongly site dependent^30^. Therefore, our ability to properly interpret this kind of archive will depend on the analysis of other analogous deposits particularly in a regional context.

## Methods

The sampled logs were sawn in half along a vertical plane parallel to the growth axis of the concretions. The thin sections of the concretions were observed with a Zeiss optical microscope and an Olympus SZ60 binocular magnifier. To make the counting and correlation of the laminae between concretions easier, the thin sections were also scanned at a 2400 dpi resolution. The counting and measurements were done under the binocular observation and from the scanned images conjointly. A linear model was then built in order to report the geochemical data as a function of the lamina number. A linear relationship between the cumulated thickness and the lamina number was calibrated on each concretion, which serves as a reference, by least-square regression. The Scilab^TM^ software was used to assign each sampling point to the corresponding laminae couplet using this linear relationship.

We used two different methods of sampling to analyze the evolutions of the isotopic signals. On the whole deposits, powders were sampled along the growth direction with a hand rotating drill. The sampling was done on successive traces, parallel to the lamination, 1 mm thick, 1 cm wide, at 0.5–2 mm intervals. Depending on the location along the growth axis, one sample may correspond to one or a few years of growth. For a finer analysis, series of samples were done on a 2 cm thick segment of S1 with a micromill Newwave Research Model 12–0223 operated by computer. The micromilling was done on parallel, successive traces 200–300 µm thick at 500 µm intervals.

The powders were heated to 90 °C for one hour and analyzed using a mass spectrometer Micromass Isoprime coupled to a Multicarb system. S1 and GL4 were analyzed at the GEOTOP laboratory in Montreal, Canada. Two internal standards were analyzed with the samples: UQ6 (δ^18^O = −1.40‰ and δ^13^C = 2.25‰ PDB) and NBS18 (δ^18^O = −23.01‰ and δ^13^C = −5.01‰). FG1 was analyzed at the ISTeP laboratory in Paris, France. The isotopic ratios of the internal standards are known with a precision better than 0.005‰. Isotope values are reported on the VPDB scale.

Sample S1 was analysed for trace elements by laser ablation inductively coupled mass spectrometry (LA-ICP-MS) at the Pôle de Spectrométrie Océan, Institut Universitaire Européen de la Mer, University of Brest. Analyses were carried out using an Excimer (193 nm Wavelength) laser ablation system (GeolasPro 102), connected to a Thermo Element XR Spectrometer. The measurements were carried out using a cross-line scan from bottom to top along the growth direction of the concretions at a scan speed of 12.5 µm/s, a laser energy of 15 J/cm^2^ and a 10 Hz pulse rate. A spot size of 50 µm was used for the first half of S1 and of 90 µm for the second half. Helium was used as a carrier gas. After the profile measurements, the background noise of the ICP-MS was measured for 100 to 200 s and subtracted to each element. The standards NIST 612 and BCR2 were analysed before each profile to calibrate the analyses. The results are displayed as element/Ca ratios. The isotopes measured were ^25^Mg, ^29^Si, ^43^Ca, ^44^Ca, ^55^Mn, ^88^Sr, ^138^Ba, ^208^Pb, ^238^U. For the first 2 cm of S1, two profiles 1–2 cm apart were carried out to check the reproducibility of the measurements. The amount of Ca measured was controlled along the successive profiles to check for zones of holes. These zones were removed from the final results using a minimal threshold of 10^7^ counts.

## Supplementary information

Supplementary Information

Table S2

Table S3

Table S4

Table S5

## Data Availability

The laminae couplet measurement, isotopes and trace elements data presented can be found as Supplementary Tables S2–S5.
