# Supplementary material for: Geochemical study of carbonate concretions from the aqueduct of Nîmes (southern France): a climatic record for the first centuries AD?
Source: Sci Rep. 2019 Mar 26;9:5209. doi: 10.1038/s41598-019-41620-4 (PMC6435730; doi:10.1038/s41598-019-41620-4)
Supplement: Supplementary file 1 — Supplementary Information [file 41598_2019_41620_MOESM1_ESM.pdf]

Geochemical study of carbonate concretions from the aqueduct of Nîmes (southern France): a climatic record for the first centuries AD?

Yacine Benjelloun*[1], Julie Carlut[1], Jean-François Hélie[2], Gilles Chazot[3], Laurence Le Callonnec[4]

1 : Institut de Physique du Globe de Paris, Sorbonne Paris Cité, Université Paris Diderot, UMR CNRS 7154, 75005 Paris, France

2 : Université du Québec à Montréal, Département des Sciences de la Terre et de l'atmosphère et centre GEOTOP, Montréal H3C 3P8, Canada

3 : Université de Brest, UMR 6538 Géosciences Océan, Institut Universitaire Européen de la Mer (IUEM) Place Nicolas Copernic, 29280 Plouzané, France

4 : Sorbonne Université, CNRS-INSU, Institut des Sciences de la Terre de Paris, ISTeP, F-75005 Paris, France

*Corresponding author: Yacine Benjelloun, yacini@wanadoo.fr

Supplementary Information

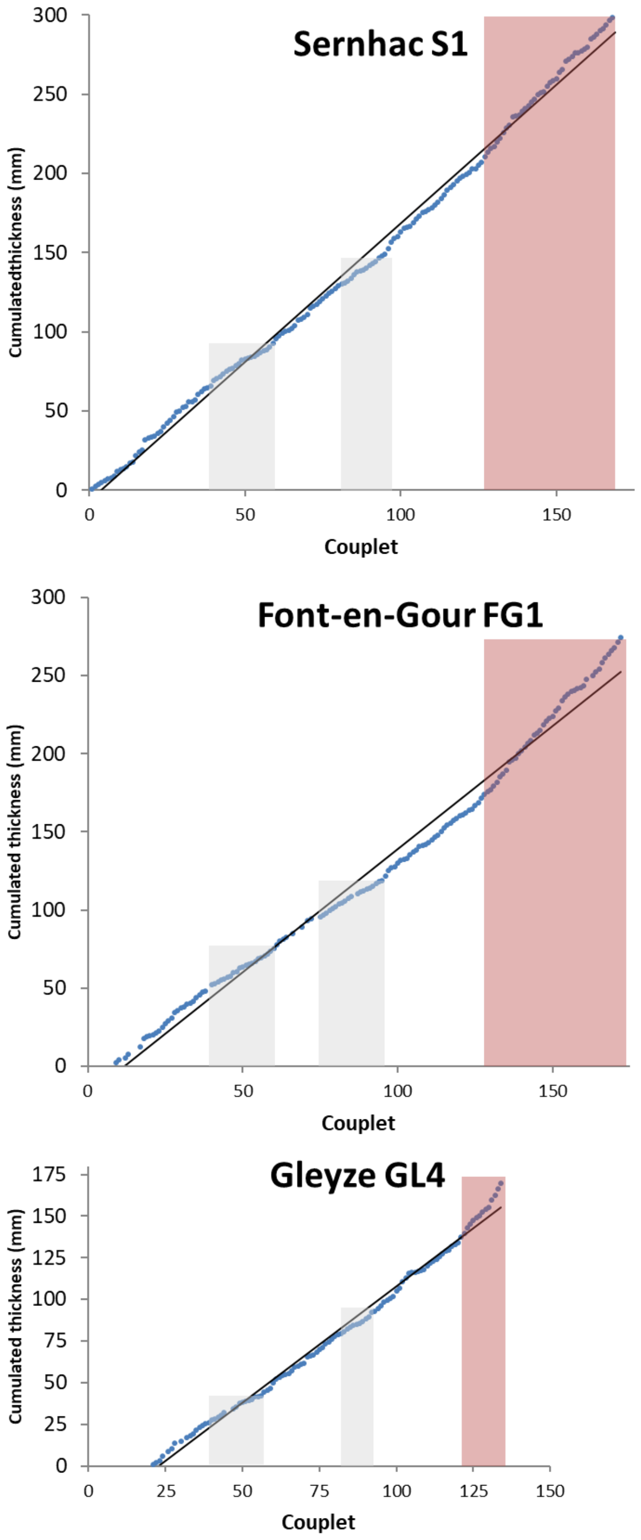

Figure S1: Cumulated laminae growth curves. The blue dots correspond to the cumulated thickness of the successive laminae couplets. The solid black line is the long-term linear trend of the series. The remarkable phases of slower and faster growth visible across the samples are indicated in grey and red respectively.

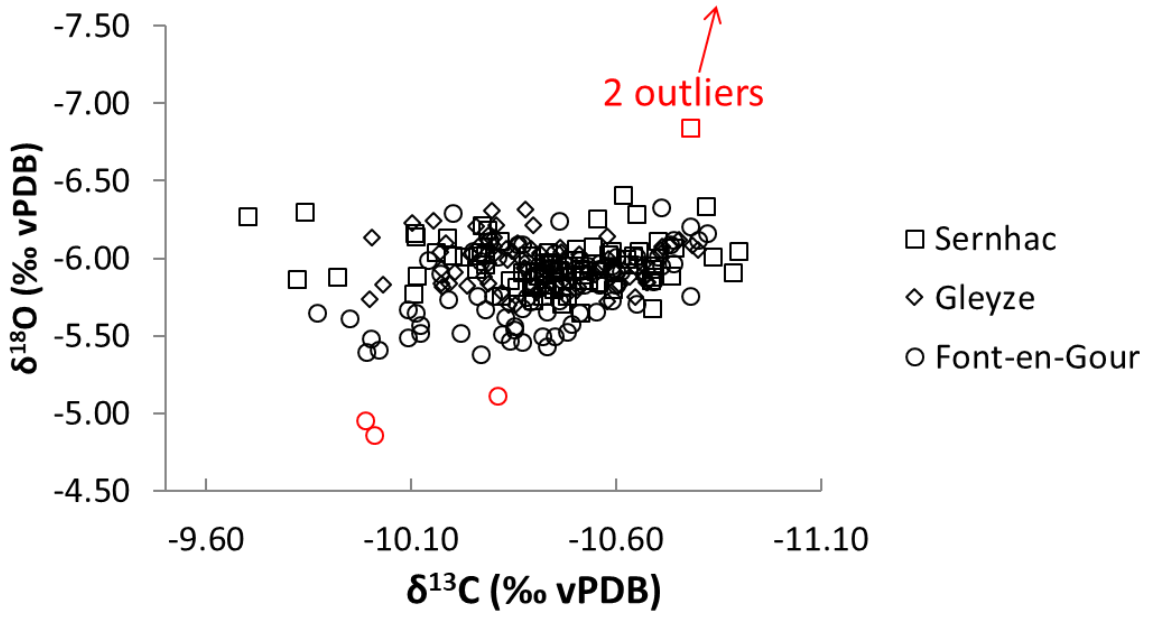

Figure S2: Comparison of the ranges of the isotopic ratios for the three samples. The $\delta^{13}C$ is indicated on the horizontal axis and the $\delta^{18}O$ on the vertical axis. The isotopic ratios are reported in ‰ against VPDB. The axes are reversed. The analytical uncertainty on the ratios is ±0.05‰ or better. The data points considered outliers and removed from the analyses and interpretations are drawn in red. Two additional outliers are way out of the range displayed and not shown.

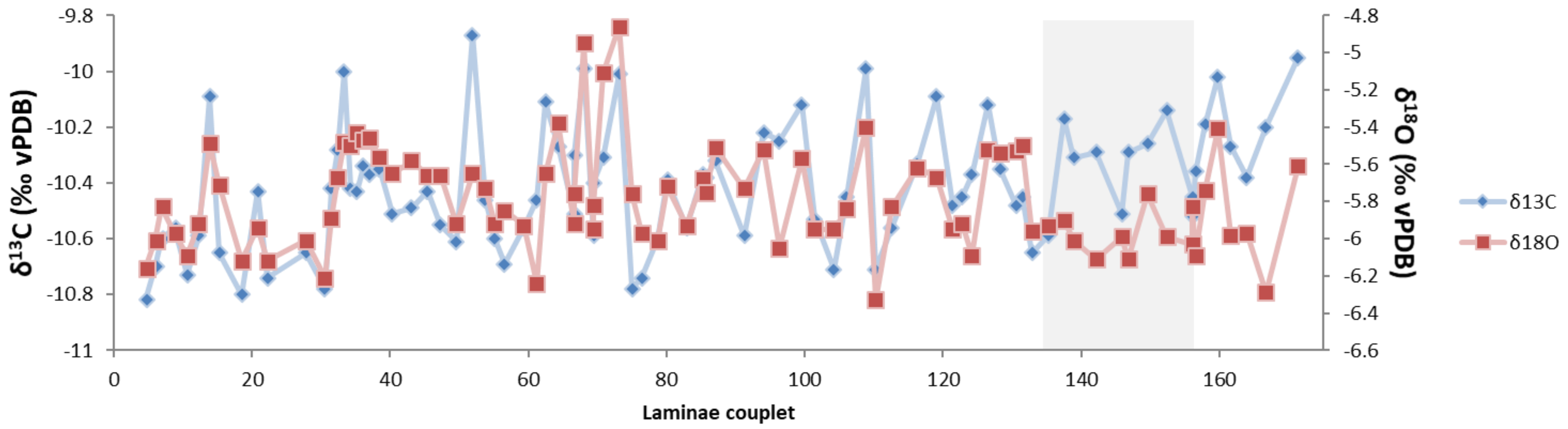

Figure S3: Compared evolution of oxygen and carbon ratios measured on sample FG1. An almost point-by-point correlation can be observed along the sample, except between laminae 135 and 155.

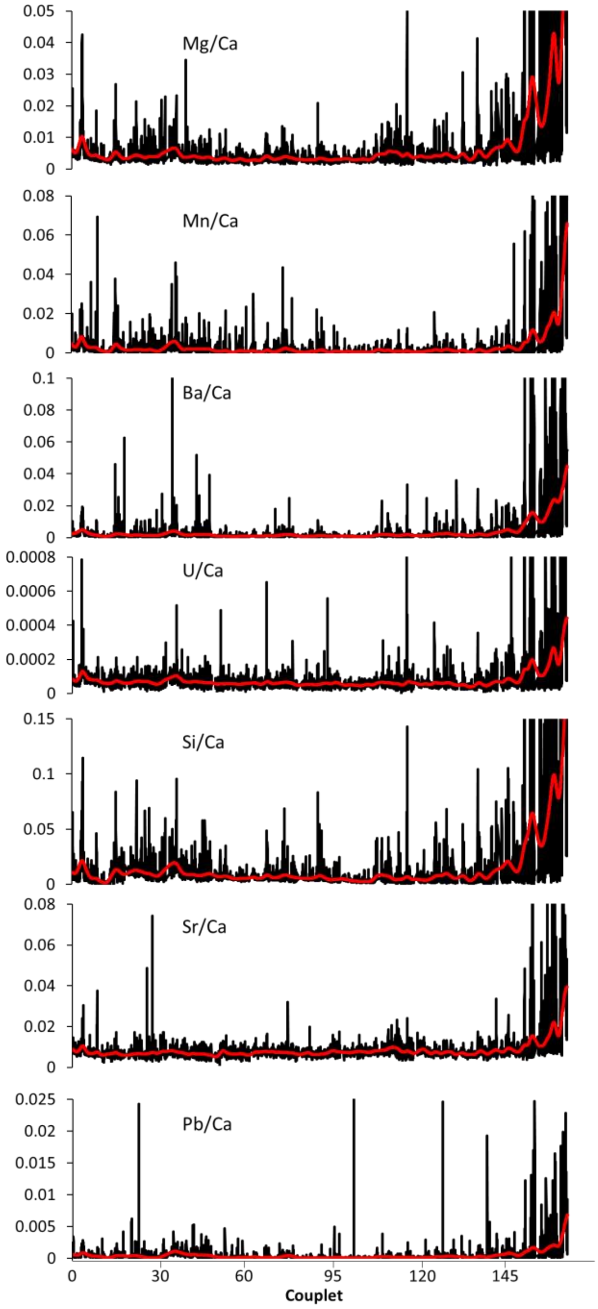

Figure S4: Raw variations of trace elements measured on Sernhac deposit. Thickness 0 corresponds to the contact with the channel wall. The smoothing applied for Fig. 8 is shown in red on some elements. The entire dataset can be found as Supplementary Table S5 online.

Location	Sample code	Coordinates (lat; lon)	Thickness (cm)
Sernhac	S1	43.904 N; 4.545 E	28
Font-en-Gour	FG1	43.896 N; 4.534 E	28
Gleyze	GL4	43.887 N; 4.528 E	22

Table S1: Location and thickness of the samples studied.
